# Materials Suitable for Osteochondral Regeneration

**DOI:** 10.1021/acsomega.4c04789

**Published:** 2024-07-02

**Authors:** Renáta Novotná, Jana Franková

**Affiliations:** † Department of Medical Chemistry and Biochemistry, Faculty of Medicine and Dentistry, Palacky University Olomouc, Hnevotinska 3, Olomouc 775 15, Czech Republic

## Abstract

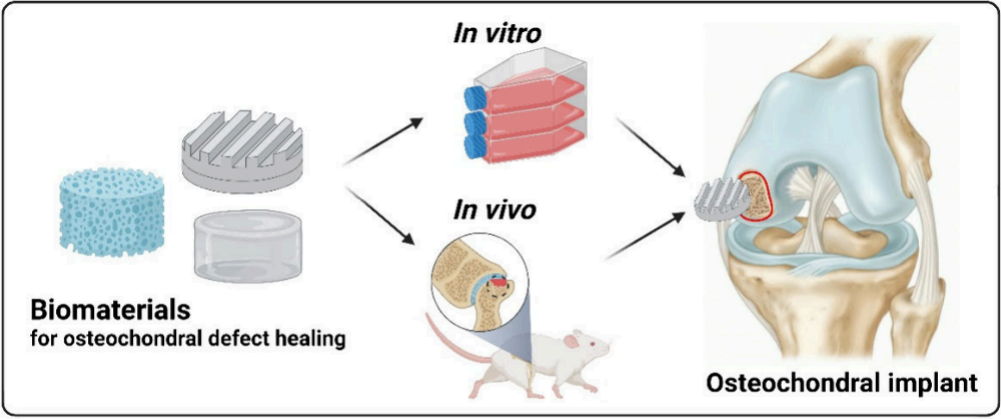

Osteochondral defects affect articular cartilage, calcified cartilage, and subchondral bone. The main problem that they cause is a different behavior of cell tissue in the osteochondral and bone part. Articular cartilage is composed mainly of collagen II, glycosaminoglycan (GAG), and water, and has a low healing ability due to a lack of vascularization. However, bone tissue is composed of collagen I, proteoglycans, and inorganic composites such as hydroxyapatite. Due to the discrepancy between the characters of these two parts, it is difficult to find materials that will meet all the structural and other requirements for effective regeneration. When designing a scaffold for an osteochondral defect, a variety of materials are available, e.g., polymers (synthetic and natural), inorganic particles, and extracellular matrix (ECM) components. All of them require the accurate characterization of the prepared materials and a number of in vitro and in vivo tests before they are applied to patients. Taken in concert, the final material needs to mimic the structural, morphological, chemical, and cellular demands of the native tissue. In this review, we present an overview of the structure and composition of the osteochondral part, especially synthetic materials with additives appropriate for healing osteochondral defects. Finally, we summarize *in vitro* and *in vivo* methods suitable for evaluating materials for restoring osteochondral defects.

## 1 Introduction

The osteochondral unit is a complex tissue region that transitions from a top layer of hyaline (articular) cartilage, through calcified cartilage into the subchondral bone layer. This tissue is found in areas of the body critical for locomotion.^1^ Osteochondral defects involve cartilage and subchondral bone and cancellous bone beneath the cartilage that form the osteochondral unit. They can be caused by injury or diseases, and are difficult to heal due to the lack of vasculature in the articular cartilage.^1,2^ These defects can occur as a partial defect where there is only injury to the cartilage layer (mostly composed of collagen fibers and GAGs), or a full-thickness defect which also includes the subchondral bone layer (mostly composed of calcium phosphate and hydroxyapatite).^1^

**Figure 1:**
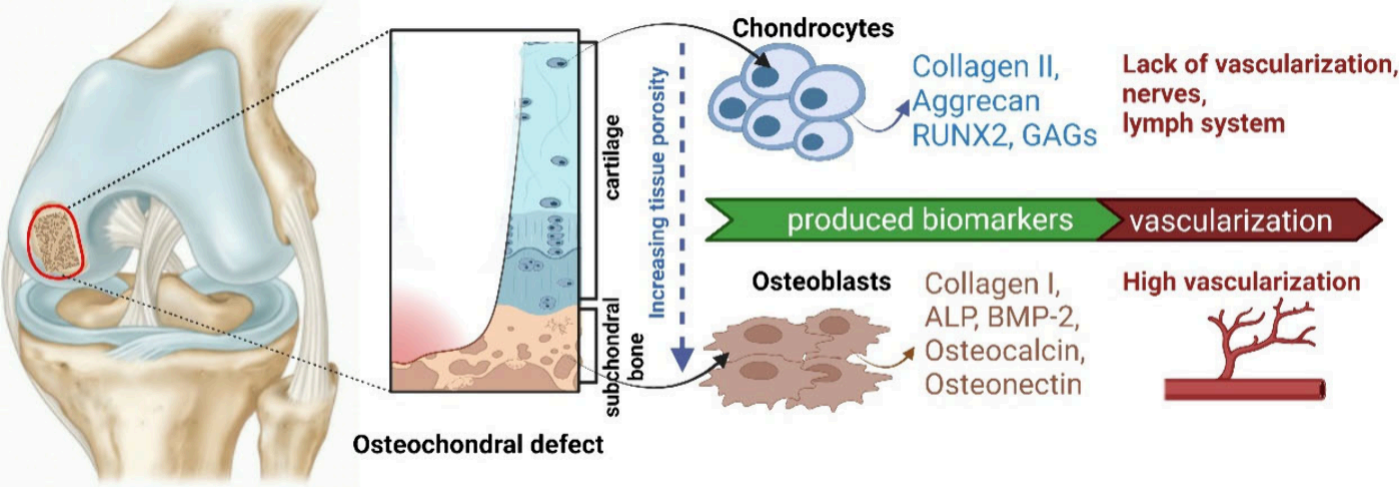
An osteochondral defect that includes both cartilage and subchondral bone damage. During healing, the main cells of cartilage–chondrocytes and bone–osteoblasts, release/express characteristic biomarkers. “Created with BioRender.com”.

The mineral content increases from cartilage to bone, while the collagen and water concentration diminish.^3^ The mechanical properties of bone differ based on the load orientation (anisotropy) and the speed at which the load is applied (viscoelasticity).^4^ Structurally, pore size, porosity, and vascularization increase from cartilage to bone Figure 1. Mechanically, compressive modulus increases from cartilage to osseous tissue.^5^

Many studies are focused on strategies for healing the subchondral unit. Due to the complicated architecture of this tissue, biphasic and multiphasic scaffolds containing two or more different layers could mimic the physiology and function of this tissue with a higher degree of similarity.^6,7^ The new materials have to reflect the native osteochondral tissue that can be a multilayer system (bilayer, trilayer) using 3D printed technologies.^8−10^ A good restorative effect can be achieved if it has a hierarchical structure, optimal porosity and mechanical properties, and contains bioactive components. A multilayer system should simultaneously meet the following requirements: (i) a biomimetic chondrogenic microenvironment and structure of the cartilage layer for supporting cartilage regeneration, (ii) a biomimetic osteogenic microenvironment and structure of the bone layer for supporting bone regeneration, and (iii) a biomimetic interface between the cartilage and bone layer similar to the native osteochondral interface.^11^

This review summarizes osteochondral defects, materials that can be used for healing these defects, and in vitro and in vivo evaluation of these materials.

## 2 Osteochondral Unit

### 2.1 Cartilage Part

Cartilage is composed of collagen (mainly type II, IX and XI), proteoglycans, GAGs and 75% water Figure 1.^12−15^ Aggrecan is a central proteoglycan carrying glycan. Aminoglycans chains retain water. The key GAGs are hyaluronic acid, chondroitin sulfate, keratan sulfate, and dermatan sulfate.^6,9,12^

Hyaline cartilage is formed of cells called chondrocytes that are surrounded by ECM composed mainly of collagen II, water, and proteoglycans. Chondrocytes are metabolically active and occupy less than 10% of the tissue. However, chondrocytes in calcified cartilage express a hypertrophic genotype and produce mainly type X collagen to the calcified ECM.^9^ The cartilage ECM is characterized by a hypoxic environment which plays a dual role in ECM regulation. First, it prevents the hypertrophy of newly formed chondrocytes, and second maintains the normal chondrocyte phenotype.^110^ There are no blood vessels or nerves in this tissue.^16^ Due to several factors including the lack of vascularization, nerves or lymph system, low mitotic activity, and low ECM turnover rates, articular cartilage rarely heals spontaneously.^17^

### 2.2 Bone Part

The bone tissue is mainly composed of collagen I,^14^ proteoglycans and their composites such as osteonectin, osteopontin, and osteocalcin, and inorganic components such as hydroxyapatite, bicarbonate, citrate, magnesium, potassium, and sodium ions Figure 1.^9^ Osteochondral bone is the final region of osteochondral tissue that separates calcified cartilage from the cement line. Osteoblasts, osteoclasts, and osteocytes are the main cells of this part, although osteoblasts promote bone formation, and their differentiation and proliferation are mediated by several factors: BMPs, TGF-β, and Runx-2 (Runt related transcription factor 2).^18^ The osteoblast differentiation process can generally be divided into three distinct stages that are defined by (1) proliferation, (2) matrix maturation, and (3) mineralization.^19^ They are differentiated from mesenchymal stem cells (MSCs), and can secrete a variety of biological active substances.^20^ Immature osteoblasts produce many specific proteins, e.g., ALP, collagen I, osteopontin, and sialoprotein, and are the cells responsible for bone formation.^21^ During differentiation, osteoblasts increase bone matrix production.^22^

However, osteoclasts are specially terminally differentiated cells derived from blood mononuclear macrophage systems,^23^ and they produce osteocyte markers, e.g., SOST, DMP1, PHEX, and MEPE, and a downregulated expression of RUNX2 and COL1A1.^24^ They adhere to the bone surface and cause local bone destruction.^25^ The main physiological function of osteoclasts is to degrade mineralized bone matrix.^26^ This involves the dissolution of crystalline hydroxyapatite and proteolytic cleavage of the calcified extracellular matrix composed of organic molecules and hydroxyapatite, which is rich in collagen.^27^

Bone resorption is necessary for many skeletal processes. It is an obligatory event during bone growth, tooth eruption, and fracture healing, and it is also necessary for the maintenance of an appropriate level of blood calcium. It is a complex process involving highly coordinated interactions between osteoblasts and osteoclasts that are modulated by a system composed of the receptor activator of nuclear factor-kappa B (RANK), the RANK ligand (RANKL), and osteoprotegerin (OPG).^27^ Osteoclasts, the cells responsible for bone resorption, can be regarded as a prototype of osteoimmune cells. Typical osteoimmune disorders (e.g., rheumatoid arthritis, osteoporosis) that are characterized by bone erosions in multiple joints in conjunction with inflammation of the synovium, are stimulated by osteoclasts.^28,111^

The resorption of bone consists of a multistep procedure requiring attachment to the bone surface, remodelling of cytoskeletal structure, polarization of the membrane, and the transport of vesicles.^29^

### 2.3 Osteochondral Defects

The development of an osteochondral defect is caused by trauma, disease, or aging.^30^ It is driven by a serious factor mainly in response to an inflammatory environment, e.g., inflammatory cytokines IL-1β, IL-6, and tumor necrosis factor α (TNF-α). The local inflammation has been proved to act as a barrier to the osteogenic and chondrogenic differentiation of MSCs.^23^ At the same time there is an increasing production of reactive oxygen species (ROS), which subsequently contribute the secretion of matrix metalloproteinases (MMP) and ADAMTS (A Disintegrin and Metalloproteinase with Thrombospondin motifs).^31,112^ Both oxidative stress and inflammation can be involved in the development of osteoporosis by preventing the differentiation of osteoblasts, inducing the differentiation and activity of osteoclasts, enhancing apoptotic osteocytes, and increasing the expression of RANKL and the RANKL/OPG-ratio.^113^ However, during the inflammation collagen II is degraded, which is the main component of hyaline cartilage, and this impairs cartilage function and increases osteoclastic activation, which reduces bone healing.^114,115^

The most important studied growth factors involved in osteochondral defects are transforming growth factor (TGF-β), bone morphology factor (BMP), insulin growth factor (IGF), and fibroblast growth factor (FGF),^9^ which are involved in bone and cartilage formation and regeneration.

The role of vasculature in bone and osteochondral development, growth and repair are well documented.^32^ Vasculature has been found to promote the expression of osteogenic genes in hypertrophic chondrocytes, thus promoting the initiation of the chondrocyte to osteoblast transformation.

Normal cartilage contains no blood vessels. In osteoarthritis, cartilage angiogenesis is involved in osteophyte development, subchondral bone remodelling, and cartilage mineralization. Osteoblasts and osteoclasts express vascular endothelial growth factor (VEGF) and its receptors, which are important during angiogenesis and bone remodelling. Blood vessel development at the osteochondral junction could increase osteochondral ossification. Pro-angiogenic factors VEGF and FGF were detected in normal cartilage (despite the fact that there are no blood vessels present).^33^ Inflammation can trigger angiogenesis.

During osteoarthritis, chondrocytes overproduce matrix metalloproteinases, and there is an increased production of collagen I and III and nonspecific alkaline phosphatase. Osteoblasts are characterized by an increased production of collagen I, interleukin 6 and 8.^33^ This part combines the main characteristic of cartilage and bone and forms an interface between them.

## 3 Materials Suitable for Healing Osteochondral Defects

Scaffolds are a crucial component of tissue engineering, because they offer a three-dimensional structure for cell adhesion and growth.^116^ Scaffolds are designed using a wide range of synthetic or natural polymers. In contrast to artificial materials and structures, biomaterials have biological traits that replicate the original type of tissue, enable favorable signal transduction, cytocompatibility, and biodegradation. Some of the materials used for regeneration are composed of a general polymer enriched with some bioactive compounds.^34^ Bioactivity was defined by Larry Hench as a material property that leads to the formation of a very strong bond between biomaterials and bone tissue.^35^ Hierarchical materials are beneficial for material–cell interaction, as they mimic the hierarchical structure of tissue. Biomaterials for the construction of multiphasic osteochondral scaffolds are generally used to make a mineral-containing layer for bone regeneration and a polymer layer for cartilage regeneration.^7,117^ The most frequently explored materials for osteochondral tissue are synthetic: e.g., polyethylenglycol (PEG), polyvinyalcohol (PVA), polyamino acid (PAA)^65^ or natural polymers, e.g., chitosan, hyaluronic acid, chondroitin sulfate, alginate, silk, and gelatin.^7,39^

### 3.1 Synthetic Polymers

Polycaprolactone (PCL) has been widely used in tissue engineering for the fabrication of bone scaffolds due to its excellent biocompatibility, slow degradation (2–3 years), and mechanical properties.^36,118^ However, it has low bioactivity and needs to be supplied with some bioactive additives, e.g., hydroxyapatite (HA), tricalcium phosphate, and bioactive glass (BG),^37^ or ceramic additives.^38^

Polypept(o)ides or poly(amino acid)s (PAA) are formed by the ring-opening polymerization of amino acid *N*-carboxyanhydrides (NCA) and have the benefits of natural and synthetic polymers. Yang et al. prepared the material PAA-RGD (thiol/thioester dual-functionalized hyperbranched polypeptide P(EG3Glu-*co*-Cys) and maleimide-functionalized polysarcosin, which promote the proliferation and chondrogenesis of MSCs and produce ostechondral repair in New Zealand Rabbits.^39^

Poly(lactide-*co*-glycolide) (PLGA) is a very useful biodegradable polymer due to its tunable biodegradation rates and very good mechanical and elastic properties,^40^ with a faster degradation period than PCL.^36^ It has been used for preparing a bilayered scaffold with a different pore size and porosities in the chondral (100–200 μm) and osseous layer (300–450 μm). The combination of PLGA and β-TCP exhibited very good biocompatibility, osteinductivity and biodegrability both in vitro and in vivo.^41^

Poly(vinyl alcohol) (PVA) is a synthetic polymer used in tissue engineering scaffolds for its ease of fabrication.^42^ Previous reports demonstrated that PVA is suitable for the fabrication of 3D-printed scaffolds with sufficient physical stability for bone tissue engineering. PVA has a unique adhesive function which can interact with components in the ECM. PVA exhibited hydrophilicity, permeability, biodegradability, and biocompatibility. It is able to retain a large amount of water or biological fluid without dissolving.^43^

Gelatin-methacrylate (GelMA), a derivate of gelatin, possesses a large number of arginine-glycine-aspartic acid sequences that are favorable for cell adhesion, migration and growth.^11,44,119^ It has excellent biocompatibility and enzymatic degradation.^45^ Pure GelMA has poor mechanical properties and a relatively rapid degradation rate.^46^ Moreover, GelMA exhibits superior printability properties compared to gelatin and other bioinks.^47^ GelMA hydrogel, prepared by optical cross-linking, has advantages such as injectability and low toxicity.^62^ Yang et al. prepared a PAA-based hydrogel (PAA-RGD) that promoted the proliferation and chondrogenesis of MSCs.^39^ Gellan gum is a calcium-cross-linkable polysaccharide hydrogel that could be used for cartilage/bone regeneration due to its structure being similar to the natural glycosaminoglycan present in cartilage, and its high affinity for calcium.^66^ Furthermore, conductive hydrogels comprise a water-soluble polymer and a network infused with conductive materials, e.g., carbon nanotubes, graphene oxide, or a conductive polymer. These materials impart electrical stimuli to the wound site and support the migration, proliferation and differentiation of cells and angiogenesis.^120^ Graphene oxide (GO) is produced by the oxidation and exfoliation of graphite. It contains abundant oxygen-containing groups such as carboxyl, epoxy, carbonyl, hydroxyl, etc.^58^ GO exhibits an efficiency for anchoring calcium ions and increasing mineralization, differentiation, and cell proliferation. GO can act as an enhancer of the mechanical, electrical and cellular properties of a hydrogel.^62^ It is able to interact with different polymers and support bone wound healing^63^ and promote bone tissue regeneration^64^ through biomineralization that accelerates the formation of calcium phosphate crystals, including HAP.^58^

### 3.2 Natural Polymers

Natural polymers are generally better than synthetic polymers in terms of cytocompatibility and bioactivity, but relatively weak in terms of mechanical properties and degradation.^121^ The commonly utilized natural polymers include: chitosan, hyaluronic acid, gelatin, collagen, silk, alginate, and cellulose.^7,9,48−51^ Chitin, collagen, and chitosan are the most widely employed natural polymers for medical applications, especially in bone tissue engineering.^9,48,51^ A previous study demonstrated that photo-cross-linked alginate hydrogels were able to repair bone defects by delivering osteogenic materials.^49^

A silk-based hydrogel with CuTa nanozyme (i.e., CuTa@SF hydrogel) combined tannic acid (TA) and copper nanoparticles.^31^ It has been reported that Ta, with its antioxidant and anti-inflammatory properties, is able to decrease the intracellular ROS level, and the copper accelerated cell proliferation, thereby promoting tissue regeneration. The materials suitable for osteochondral regeneration are summarized in Table 1.

**Table 1:** Summary of Materials Used for Healing Osteochondral Defects Included in This Review^a^

materials	osteochondral part	cells/animal	methods
PCL/PLA/PLGA 85:15	bone	MSCs	biocompatibility
PLGA 65:35	cartilage	mice	histological staining
1.5% alginate/agarose			mineral content^36^
PCL/BBG	bone	MSCs	cytocompatibility
borate fraction: 0%, 5%, 10%, 20% a 40%		rabbit	histological and fluorescent staining
			micro-CT^37^
polysyntheticpolypeptide	bone	MSCs, RAW246.7	biocompatibility
P(EG_3_Glu-*co*-Cys)		rabbit	histological staining
			qRT-PCR
			micro-CT^39^
PLGA	cartilage	BMSCs	biocompatibility
		rabbit	histological and fluorescent staining
			RT-PCR^40^
PLGA/KGN-PLGA/β-TCP/CIN	bone	rabbit	histological and fluorescent staining
lactide/glycolide 75:25	cartilage		micro-CT^41^
CIN 0.5%, KGN 1%			
alginate/silk fibroin/PVA	bone	MC3T3E1	biocompatibility
alginate/PVA 3:1			histological staining^42^
silk fibroin 0%, 0.5%, 1% a 2%			
ZrO_2_-GM/SA	bone	MC3T3E1	biocompatibility
GelMA 5% SA 1%		mice	histological and fluorescent staining^45^
GelMA 5% SA 2%			
GelMA 10% SA 1%			
GelMA 10% SA 2%			
^USC^GelMA-HAMA/nHAP	bone	BMSCs	biocompatibility
- GelMA 5%, HAMA 1%, nHAP 0.5%		rat	histological staining
- GelMA 5%, HAMA 1.5%, nHAP 0.5%			micro-CT^46^
- GelMA 5%, HAMA 2%, nHAP 0.5%			
GG	bone	MG63	biocompatibility^66^
GG 0.7%, 1%, 1.3%	cartilage		
with ALP 0.1%, 0.5% and 5%			
GO/HAp	bone	MC3T3E1	biocompatibility^58^
HA/SA 4:1	bone	BMSCs	biocompatibility
		rat	PCR
			histological and fluorescent staining^49^
β-TCP	bone	J774.2	biocompatibility^52^
		RAW264.7	
BG-TCP	bone	BMSCs	biocompatibility
		rat	histological and fluorescent staining
			micro-CT^54^
β-TCP	bone	human	histological evaluation
			radiographic images^55^
chitosan/PVA/nano BG/nano ZnO	bone	osteoblasts	biocompatibility
chitosan/PVA 1:1			antibacterial activity^43^
ZnO 0.05%, 0.1%, 0.2%			
HAp/PVA	bone	BSMCs	biocompatibility^19^
gelatin/PLLA	bone	L929	biocompatibility^60,61^
Osteogenon 1.5%, 3% and 5%	cartilage		
collagen II	bone	rat	histological and fluorescent staining
	cartilage		micro-CT
			gene expression^122^
MACS	bone	BMSCs	biocompatibility
	cartilage	pig	histological assessment
			micro-CT^123^
Sr-ACP granules in Col/Col-Mg-HAp	bone	Balb/3T3	biocompatibility
	cartilage	mice	histological assessment
		goat	micro-CT^124^
GelMA/HA	bone	ADSCs	biocompatibility
	cartilage	rabbit	histological assessment
			micro-CT^125^
Coll I/HA	bone	goat	histological assessment
Coll 0.25%, HA 0.05%	cartilage		micro-CT^126^
CM-KGN@GelMA	bone	BMSCs	biocompatibility
KGN 0.1 mg/mL	cartilage	rat	histological assessment
GelMA 50 mg/mL			micro-CT^127^
GelMa/HA	bone	MSCs	biocompatibility
GelMA 5%	cartilage	mice	histological assessment^128^
HA 2%			
BG-TCP 0%, 5%, 15%, 25%	bone	SMSCs	biocompatibility
		EPCs	histological and fluorescent staining
			micro-CT
			qRT-PCR^129^

a PCL, polycaprolactone; PLA, polylactic acid, poly(lactic-*co*-glycolic acid); BBG, borate bioactive glass; KGN, kartogenin; CIN, cinnamaldehyde; TCP, tricalcium phosphate; PVA, poly(vinyl alcohol); GM/SA, GelMA (gelatin methacrylate) and sodium alginate; HA, hyaluronic acid; HAMA, hyaluronic acid methacrylate; GG, gelan gum; GO, graphene oxide; HAp, hydroxyapatite; HA/SA, hyaluronic acid and sodium alginate; BG, bioactive glass; PLLA, polylactide; MACS, methacryloyl chondroitin sulfate; Sr-ACP, strontium amorphous calcium phosphate; CM, cytomodulin; P(EG_3_Glu-*co*-Cys), copolymerer of γ-(2-(2-(2-methoxyethoxy) ethoxy) ethyl l-glutamate NCA (L-EG_3_GluNCA); and l-cysteine NCA (CysNCA).

### 3.3 Other Components of Scaffolds

#### 3.3.1 Tricalcium Phosphate

β-Tricalcium phosphate (β-TCP) is a synthetic ceramic with a chemical composition close to the mineral phase of bone.^52^ It was reported that TCP has osteoinductive properties and bioresorbality, and provides a reasonable template for the formation of new bone.^53^ Several studies have investigated the potential of bioactive glass for improving the properties of β-TCP ceramics, including high porosity and interconnectivity, favorable pore shape, appropriate pore size, sufficient mechanical strength and superior bioactivity.^54^ A difference in β-TCP porosity was reported to influence its mechanical durability and the length of time until artificial bone blocks are replaced with native bone tissue.^55^

#### 3.3.2 Bioactive Glass

Bioactive glass (BG) is a ceramic material that can be incorporated into the polymer matrix to create a nanocomposite with high potential for biomedical application. It is able to form a bond between soft and hard tissue and enhance stiffness.^119^ Its great osteoinductivity and osteoconductivity to stimulate osteoblast activity has been demonstrated.^43^ Mesoporous bioactive glass is extensively studied in bone healing due to its good biocompatibility and bioactivity—in vitro mineralization.^8^ Controlled ion release from bioglass is critical for osteogenesis. Also, the cellular activity of ALP (an early marker of osteoblast differentiation) increases with the amount of bioglass.^35^ The pore size of bioglass loaded with BMP-2 is in the range of 300–500 μm, which is important for mineralization, due to the large amount of calcium-phosphate deposition.^56^ Elements such as calcium, phosphorus, and magnesium liberated from the bioceramic composition create a microenvironment that is similar to the native microenvironment in situ, thus stimulating bone reconstruction and neoangiogenesis.^54^

#### 3.3.3 Hydroxyapatite

The mineral hydroxyapatite (HAP) is naturally synthesized and comprises 70% of the skeleton by weight and 50% by volume,^4^ and 90% by weight of tooth enamel. HAP consists mainly of calcium and phosphate Ca_5_(PO_4_)_3_(OH) (or more often referred to as the two units’ crystal form: Ca_10_(PO_4_)_6_(OH)_2_) in a ratio of 1.67), and is crystalline in form.^57^ HAP increases the local concentration of calcium that activates osteoblast proliferation.^130^ Nanohydroxyapatite (n-HAP) breaks down in a decomposition process to produce water and organic and inorganic compounds essential for bone healing.^4^ Nanosized hydroxyapatite, which is generated endogenously by osteoblasts in the form of matrix vesicles as the initiator of bone formation in the skeleton, has great osteoinductive properties.^131^ As well as playing a role in the pathological calcification of cartilage and vasculature, it can be deposited in soft tissues in the form of dystrophic and metastatic calcifications.^19^ n-HAP is a bioactive compound that can form a strong bond with bone, it is able to deposit in bone and react with proteins, resulting in an osteogenic process.^58^ However, the available results indicate that pure HAP has poor mechanical properties and cannot be used as a load-bearing implant material, which is forcing materials scientists to search for HAP composites with high load-bearing capacity.^59^ The combination of ossein and hydroxyapatite forms a complex with osteocalcin and type collagen I^60^ (Osteogenon, Osteo, Pierre Fabre). This bioactive material supports cell proliferation, adhesion and bone mineralization.^61^ It has two effects on metabolism: it inhibits osteoclasts and stimulates osteoblasts. It has been applied in tablet form for its analgesic effect and for reducing bone loss and the chance of fracture in patients with secondary osteoporosis. It is more effective at preventing bone loss than other calcium salts.

### 3.4 Fabrication Technique

Fabrication technique plays a crucial role in the development of new materials, and consequently has an influence on repair outcomes by modulating biological responses through controlling pore structure, mechanical properties, the spatial distribution of materials and/or additives such as cells and growth factors, and biodegradation. Currently utilized methods employed for osteochondral scaffolds to achieve multilayer or gradient designs include electrospinning, 3D printing, lyophilization, freeze casting, gas foaming, microfluidic foaming, the sol–gel process, melt molding, compression molding, particulate leaching, phase separation, and additive manufacturing.^132−135^

Extrusion printing is the most commonly used method for constructing 3D architecture in osteochondral tissue engineering studies because of its relatively low cost, high availability, and ease of use.^141^ 3D printing technology, also known as additive manufacturing, allows precise control over the composition and spatial distribution of cells and biomaterials to create scaffolds for tissue repair and regeneration.^51^ It involves stacking layers of biologically active materials filled with cells and growth factors to create highly biomimetic tissue microenvironments, structures, blood vessels, and functional artificial organs.^138^ Evolving 3D bioprinting approaches have resulted in a number of different printing strategies, including free-form reversible embedding printing of suspended hydrogels, extrusion-based multimaterial point-dispensing printing, void-free 3D bioprinting, and layer-by-layer alternating bioprinting with a double mesh for tissue analogues. As tissues contain multiple structures and diverse cells, and cell phenotype is sensitive to the biochemical and mechanical properties of the microenvironment, bioinks for 3D bioprinting are becoming increasingly important for the generation of multiple structures and diverse cell-engineered tissues. This enables the production of patient-specific tissues with the correct size and shape.^13,139,140^

## 4 Evaluation of Materials for the Treatment of Osteochondral Defects: *In Vitro* and *In Vivo* Methods

**Figure 2:**
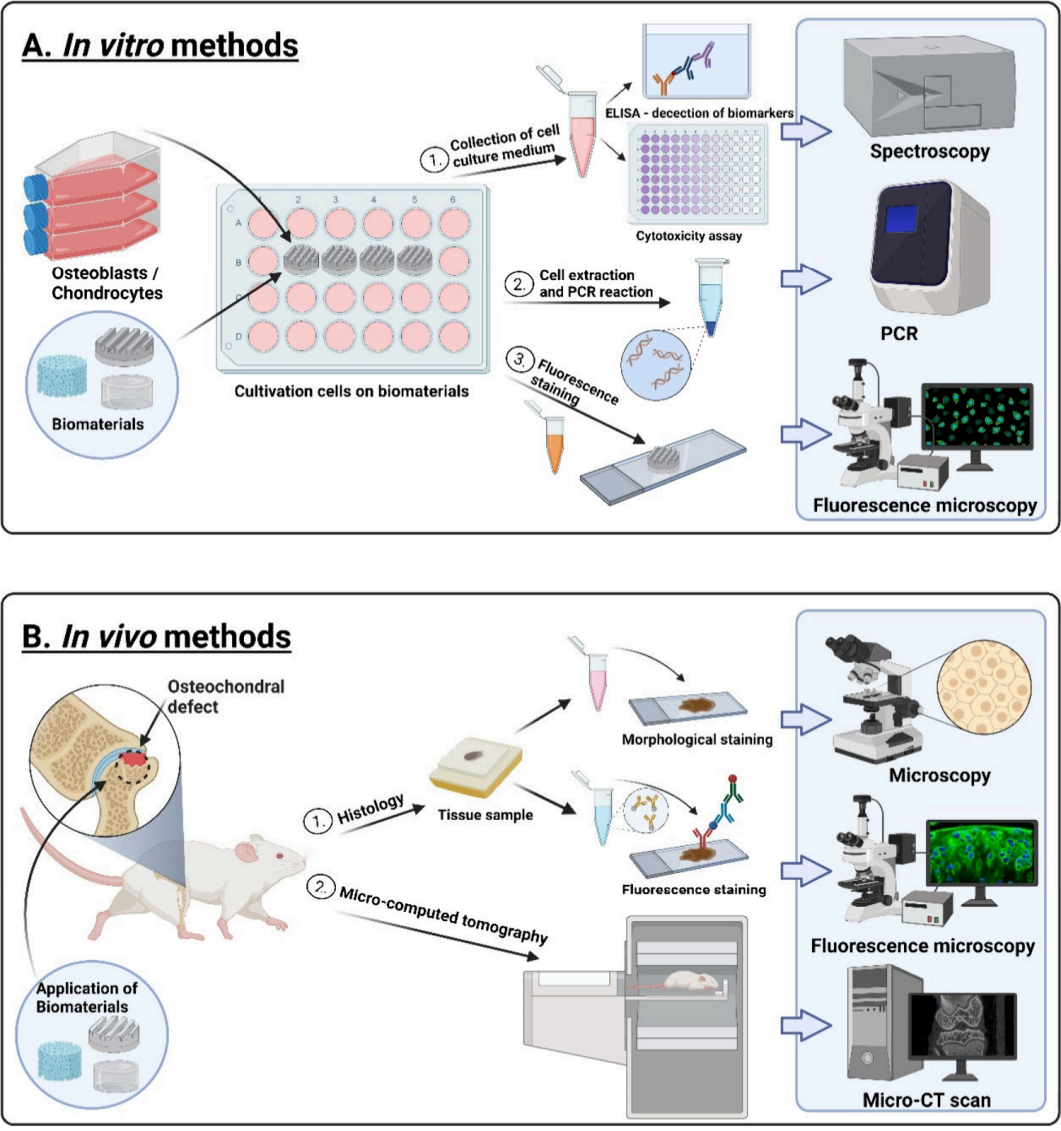
(A) *In vitro* assessment of biomaterials (determination of cytotoxicity, biocompatibility and selected biomarkers) and (B) evaluation of biomaterials by *in vivo* methods, using histological staining and micro-CT. “Created with BioRender.com”.

This review aims to present the most widely used methods for evaluating materials for the treatment of osteochondral defects. Special attention will be paid to *in vitro* (e.g., cytotoxicity, biocompatibility) and *in vivo* methods (e.g., histological staining, CT etc.), which enable systematic and comprehensive testing of these biomaterials in the laboratory and clinical environment (Figure 2).

### 4.1 *In Vitro* Methods

First of all, it is important to determine cytotoxicity and biocompatibility in vitro. There are several established methods for this:

#### 4.1.1 MTT Test

(3-(4,5-Dimethylthiazol-2 yl) 2,5-diphenyltetrazolium bromide): this staining method measures cell viability in the presence of a biomaterial. Cellular mitochondria convert MTT to a purple formazan precipitate, which can be quantified spectrophotometrically.^8,67^

#### 4.1.2 LDH Test

(Lactate dehydrogenase): this measures the release of the LDH enzyme from cells, which is an indicator of cell damage. Elevated LDH levels indicate the cytotoxicity of a biomaterial.^68^

#### 4.1.3 AK Test

(Adenylate kinase): the release of adenylate kinase from cells can be an indicator of cell damage and cytotoxicity caused by a biomaterial.^69^

#### 4.1.4 Live/Dead Staining

Fluorescent dyes such as acridine orange, fluorescein diacetate, and propidium iodide are used to visualize live and dead cells on the surface of a biomaterial under a microscope.^8,70^

##### 4.1.4.1 Alexa Phalloidin Cytoskeleton and DAPI Nuclear Staining

Alexa phalloidin cytoskeleton and DAPI nuclear staining are used to determine cell adhesion, proliferation and biocompatibility on biomaterials.^8,71^

#### 4.1.5 Alkaline Phosphatase (ALP)

ALP is an enzyme associated with the differentiation of osteogenic cells, and plays a key role in bone mineralization processes and has a critical function in the formation of hard tissue.^72^ Higher ALP activities were indicated in cells cultured on the biomaterial, demonstrating a positive effect of the biomaterial on osteogenesis.^73^ During the osteogenic differentiation process, the presence of ALP activity indicates the differentiation of mesenchymal stromal cells (MSCs) into osteoblasts.^74^

This can be determined spectrophotometrically, by RT-PCR, Western blot, or immunostaining,^75−77^

#### 4.1.6 Bone Morphogenetic Proteins (BMPs) and TGF-β Family

BMPs are multifunctional growth factors that belong to the transforming growth factor beta (TGF-β) superfamily.^78^ TGF-β is a multifunctional chondroblast growth factor that promotes the secretion of collagen II and proteoglycans. It also plays a critical role in maintaining of homeostasis between subchondral bone and articular cartilage.^138^ BMPs are involved in several events during bone morphogenesis, including bone remodelling, bone formation, chondrogenesis, and mesenchymal cell infiltration and proliferation.^79,80^ It is mostly determined by ELISA, RT-PCR, or immunocytochemistry.^81^

#### 4.1.7 Osteocalcin (OC)

OC is a calcium-binding protein, and is the most abundant noncollagenous protein in bone.^82,83^

OC is a marker of osteoblast mineralization, and can reflect their activity. Its determination is important for assessing the ability of biomaterials to support osteogenesis. Like other osteogenic factors, it can be determined using RT-PCR and immunocytochemical staining or ELISA.^84−86^

#### 4.1.8 Runt-Related Transcription Factor 2 (RUNX2)

Runx2 is a master gene of osteoblast differentiation and bone formation.^87^ It is most often determined using RT-PCR^88^ or immunocytochemistry.^89^

#### 4.1.9 Collagen I/Collagen II

Numerous collagen subtypes have been identified in articular cartilage, such as types I and II. Collagen type I is the main component in connective tissues^90^ and is also the main organic component of the bone matrix produced by osteoblasts. The presence of type I collagen is necessary for the formation and proper regeneration of bone tissue around an osteochondral defect. In contrast, collagen type II is produced in cartilage by chondrocytes, and is a key factor in restoring the structure and function of cartilage tissue.^91^ They are most often determined using the ELISA method,^92^ RT-PCR^93^ or immunocytochemistry.^94^

#### 4.1.10 Aggrecan and SOX9

Aggrecan and SOX9 are the main markers in cartilage that characterize chondrogenic differentiation during cartilage formation. Aggrecan contains several highly sulfated GAG side chains that confer a high negative fixed charge density to the tissue, providing hydration and compressive stiffness. The amount of aggrecan/SOX9 produced by chondrocytes can be detected by RT-PCR or immunocytochemistry.^93,95−100,121,140^

Once the *in vitro* (Figure 2A) tests are completed, it is important to perform an *in vivo* (Figure 2B) evaluation of the biomaterial for the treatment of osteochondral defects. Here are some possible methods for the *in vivo* assessment of materials.

### 4.2 *In Vivo* Methods

#### 4.2.1 Histological Staining

Histological staining is a method that highlights important features of the tissue.^101^ The tissue sections are stained with hematoxylin and eosin for nuclear morphology and nonspecific tissue visualization, alcian blue to assess sulfated glycosaminoglycans content, picrosirius red to assess collagen content, and alizarin red to assess calcification.^102^

Another histological method is immunohistochemistry, which demonstrates the presence of specific proteins for osteogenesis or chondrogenesis (e.g., collagen I, collagen II, osteocalcin, ALP, aggrecan), which are influenced in various ways after the implantation of osteochondral scaffolds.^103−105^

#### 4.2.2 Microcomputed Tomography (micro-CT) Analysis

Micro-CT is a nondestructive X-ray preclinical imaging method that can scan specimens to create 2D pixel maps in thin slices through an entire object.^102,106−108^

3D evaluation is carried out on the segmented images to determine bone volume and density and to reconstruct a 3D image.

Micro-CT images are further analyzed using ImageJ to determine trabecular thickness (which quantifies bone growth), bone volume to total volume ratio, porosity, and the degree of anisotropy within the tested samples, offering insight into the regeneration of the subchondral bone.^109^

## 5 Therapeutic Application and Commercialization of the Product

Currently, the repair of osteochondral defects is one of the most difficult challenges in medicine. Before a potential treatment is applied to humans, all the necessary preclinical experiments need to be conducted (both in vitro and in vivo). In the subsequent clinical evaluation, there is a set of ongoing activities that use scientifically sound methods for the assessment and analysis of clinical data to validate safety, clinical performance, and effectiveness.^136^ For clinical applications, it is essential to include convenience, effectiveness and minimize trauma during the application process. Thus, the choice of biomaterials is extremely important and needs to be not only considered for its chemical composition, but also its physical properties.^137^

## 6 Conclusions and Future Outcome

This review summarizes materials suitable for osteochondral regeneration, and the second part of this review is concerned with in vitro and in vivo methods necessary for the evaluation of new materials and therapeutic applications. Ideally, autologous tissues (autografts) are used for medical applications, which eliminates the immunogenic response and is optimal for cell growth. Alternatively, grafts from donors (allographs) can be used, but there is the risk of immunoreaction and infection. Furthermore, these strategies are based on cellular techniques, and these products were considered as medical devices and biological medicines that need time-consuming and expensive authorization. Thus, nowadays, the majority of materials for osteochondral regeneration usually consist of synthetic or natural polymers. Some of these polymers have low biological activity, and need to be supplemented with substances with higher biological potential, e.g., β-TCP, GO, etc. Due to a wide range of biocompatibilities and biodegradabilities, these could have a different influence on the surrounding tissue and potential inflammation.

Osteochondral tissue is a complex structure with multiple hierarchies. The new material has to incorporate characteristic properties of each part of osteochondral units, e.g., porosity, the production of ECM proteins (collagen I for the bone part and collagen II for the cartilage part). The regeneration of a seamless gradient between the innervated, vascularized, and mineralized bone, and the avascular, nonmineralized, and aneural cartilage should be one of the most important tasks in osteochondral tissue regeneration. Given the diverse material composition of the indigenous osteochondral tissue, it is essential to select suitable biomaterials for each layer. Due to the complicated structure of the osteochondral part, it is difficult to find materials that perfectly substitute for a defect. This is a reason why multilayered systems are so popular. However, gradient scaffolds due to their different porosities mimic the hierarchy of the natural tissue, which plays an essential role in nutrient and oxygen transport, cell adhesion, and migration and vascular growth.

The following are recommended for the preparation of new materials:

Improving the biocompatibility of scaffolds.

Aiming for good mechanical properties and an ideal biodegradation rate.

Focusing on promising 3D-printed methods that can match the shape of the damaged part.

Optimizing transplantation (preclinical studies) to avoid the rejection of materials.

So, the major challenges are to find acellular materials that reflect the osseous and chondrogenic part, mimic the gradient microstructure, have appropriate mechanical properties, an appropriate degradation profile, and include all the requirements for healing an osteochondral defect.
